# Changes of TSH-Stimulation Blocking Antibody (TSBAb) and Thyroid Stimulating Antibody (TSAb) Over 10 Years in 34 TSBAb-Positive Patients with Hypothyroidism and in 98 TSAb-Positive Graves' Patients with Hyperthyroidism: Reevaluation of TSBAb and TSAb in TSH-Receptor-Antibody (TRAb)-Positive Patients

**DOI:** 10.1155/2012/182176

**Published:** 2012-05-10

**Authors:** Nobuyuki Takasu, Mina Matsushita

**Affiliations:** Department of Endocrinology and Metabolism, Aizawa Hospital, 2-5-1 Honjo, Mtasumoto 390-8521, Japan

## Abstract

Two TRAbs: TSBAb and TSAb. TSBAb causes hypothyroidism. TSAb causes Graves' hyperthyroidism. TSBAb and TSAb block TSH-binding to cells as TRAb, measured as TSH-binding inhibitory immunoglobulin (TBII). We reevaluate TSBAb and TSAb. We studied TSBAb, TSAb, and TBII over 10 years in 34 TSBAb-positives with hypothyroidism and in 98 TSAb-positives with hyperthyroidism. Half of the 34 TSBAb-positives with hypothyroidism continued to have persistently positive TSBAb, continued to have hypothyroidism, and did not recover from hypothyroidism. Ten of the 98 TSAb-positives with hyperthyroidism continued to have positive TSAb and continued to have hyperthyroidism. TSBAb had disappeared in 15 of the 34 TSBAb-positives with hypothyroidism. With the disappearance of TSBAb, recovery from hypothyroidism was noted in 13 (87%) of the 15 patients. TSAb had disappeared in 73 of the 98 TSAb-positives with hyperthyroidism. With the disappearance of TSAb, remissions of hyperthyroidism were noted in 60 (82%) of the 73. Two of the 34 TSBAb-positives with hypothyroidism developed TSAb-positive Graves' hyperthyroidism. Two of the 98 TSAb-positive Graves' patients with hyperthyroidism developed TSBAb-positive hypothyroidism. TSBAb and TSAb are TRAbs. TSBAb-hypothyroidism and TSAb-hyperthyroidism may be two aspects of one disease (TRAb disease). Two forms of autoimmune thyroiditis: atrophic and goitrous. We followed 34 TSBAb-positive patients with hypothyroidism (24 atrophic and 10 goitrous) over 10 years. All of the 10 TSBAb-positive goitrous patients recovered from hypothyroidism and 19 (79%) of the 24 TSBAb-positive atrophic patients continued to have hypothyroidism.

## 1. Introduction

There are two types of TSH receptor antibodies (TRAbs): thyroid stimulating antibody (TSAb) [1, 2] and TSH-stimulation blocking antibody (TSBAb) [3]. TSAb stimulates the thyroid and causes Graves' hyperthyroidism. TSBAb blocks TSH-stimulation of the thyroid and causes hypothyroidism. Both TSAb and TSBAb block TSH-binding to thyroid cells as TSH-receptor antibody (TRAb), which has been measured as TSH-binding inhibitory immunoglobulin (TBII) [1–3]. TBII indicates the inhibition of TSH-binding to TSH receptor but does not indicate the function of TRAb. TRAb can be stimulatory or inhibitory. To know whether TRAb is stimulatory or inhibitory, TSAb and TSBAb have been measured [1–3]. TRAb has been measured by different assay methods and given various names. Among them, TBII [1, 4, 5] and TSAb [1, 2, 6–9] have been measured as TRAb to diagnose Graves' disease and to follow the patients. TBII is measured as a receptor assay. TSAb is measured as a stimulator assay, using porcine thyroid cells. TSAb indicates the stimulation activity of TRAb. TSBAb [3, 10–13] and TBII [3, 4, 10–13] have been measured as TRAb to diagnose TSBAb-positive hypothyroidism and to follow the patients. TSBAb has been measured as a TSH-stimulation blocking assay, using porcine thyroid cells [3, 10–13]. TSBAb indicates the inhibitory activity of TRAb. TSAb and TSBAb are TSH-receptor antibodies (TRAb). The former TRAb (TSAb) is a stimulating antibody [1, 2, 6–9], and the latter TRAb (TSBAb) is a blocking antibody [3, 10–13]. TSBAb blocks TSH-stimulation of the thyroid and causes hypothyroidism. TSBAb blocks TSH-binding to thyroid cells and is TRAb. TSBAb blocks TSH-stimulation of the thyroid and is measured as inhibition of TSH-stimulated cAMP synthesis of thyroid cells. TSBAb and TSAb are TRAb. TBII reflects TSBAb- and TSAb-activities.

TSAb stimulates the thyroid and causes Graves' hyperthyroidism. Treatment with antithyroid drugs (ATDs) decreases serum TSAb [14]. With the disappearance of TSAb, remissions of Graves' hyperthyroidism have been seen [14]. TSBAb blocks TSH-stimulation of the thyroid and causes hypothyroidism [3]. With the disappearance of TSBAb, recovery from hypothyroidism occurs [3].

It has been generally believed that Graves' patients have TSAb but do not have TSBAb, and that blocking antibody-(TSBAb-) positive patients with hypothyroidism have TSBAb but do not have TSAb. However, TSBAb-positive patients with hypothyroidism and TSAb-positive Graves' patients with hyperthyroidism could have both TSBAb and TSAb [13]. Some patients may have TSBAb and TSAb simultaneously or sequentially [13]. The balance of TSBAb and TSAb determines whether a patient has hypothyroidism or hyperthyroidism [13]. We have encountered TSBAb-positive patients with hypothyroidism, who developed TSAb-positive Graves' hyperthyroidism, and also TSAb-positive Graves' patients with hyperthyroidism, who developed TSBAb-positive hypothyroidism. Thyroid function can oscillate between hypothyroidism and hyperthyroidism as TSBAb or TSAb becomes dominant.

There are two forms of autoimmune thyroiditis: atrophic autoimmune thyroiditis and goitrous autoimmune thyroiditis [3]. It has become evident that hypothyroidism may occur as a result of the production of TSBAb. TSBAb has been said to cause hypothyroidism in the patients with atrophic autoimmune thyroiditis [3]. However, TSBAb has been found in patients with atrophic autoimmune thyroiditis, and also in patients with goitrous autoimmune thyroiditis [11]. TSBAb was detected in 25% of the patients with atrophic autoimmune thyroiditis and in 9% of those with goitrous autoimmune thyroiditis [3]. TSBAb causes hypothyroidism. With the disappearance of TSBAb, recovery from hypothyroidism has been reported [3]. Here, we followed 24 TSBAb-positive hypothyroid patients with atrophic autoimmune thyroiditis and 10 TSBAb-positive hypothyroid patients with goitrous autoimmune thyroiditis over 10 years. All of the 10 TSBAb-positive patients with goitrous autoimmune thyroiditis recovered from hypothyroidism and 19 (79%) of the 24 TSBAb-positive patients with atrophic autoimmune thyroiditis continued to have hypothyroidism.

We reevaluated TSBAb and TSAb in TRAb-positive patients. We studied serial changes of TSBAb and TSAb over 10 years in 34 TSBAb-positive patients with hypothyroidism and in 98 TSAb-positive Graves' patients with hyperthyroidism. With persistently positive TSBAb, recovery from hypothyroidism was not observed. With persistently positive TSAb, remissions of Graves' hyperthyroidism were not obtained. With the disappearance of TSBAb, recovery from hypothyroidism was seen. With the disappearance of TSAb, remissions of Graves' hyperthyroidism were also seen. Two of the 34 TSBAb-positive patients with hypothyroidism developed TSAb-positive Graves' hyperthyroidism. Two of the 98 TSAb-positive Graves' patients with hyperthyroidism developed TSBAb-positive hypothyroidism. TSBAb-positive hypothyroidism and TSAb-positive hyperthyroidism may be two aspects of one disease (TRAb disease).

## 2. Subjects and Method

### 2.1. Subjects

 We studied 34 TSBAb-positive patients with hypothyroidism and 98 TSAb-positive Graves' patients with hyperthyroidism (Table 1). The 34 TSBAb-positive patients with hypothyroidism were treated with thyroxine (T4) and the 98 TSAb-positive Graves' patients with hyperthyroidism were treated with antithyroid drugs (ATDs). Serial changes of TSBAb and TSAb over 10 years were studied in 34 TSBAb-positive patients with hypothyroidism (I) and in 98 TSAb-positive Graves' patients with hyperthyroidism (II). TSBAb-positive patients with hypothyroidism were diagnosed on the basis of the history, signs of hypothyroidism, and the laboratory findings, including positive TSBAb (>+40%) and decreased serum-free thyroxine (fT4) and free triiodothyronine (fT3) with high TSH [3, 13]. The diagnosis of goitrous autoimmune thyroiditis was based on the finding of palpable goiter and that of atrophic autoimmune thyroiditis on the absence of goiter [3]. The 34 TSBAb-positive patients with hypothyroidism were treated with thyroxine (T4). Thyroxine was discontinued at 3 months after the disappearance of TSBAb. After the discontinuation of T4, the patients had been seen every 1–3 months. When the patients continued to be in euthyroid states and to have negative TSBAb and negative TBII for more than 1 year after the T4-discontinuation, they were considered to have recovery from hypothyroidism; otherwise, they had recurrence [3]. When serum TSH became higher than 10 mIU/L, T4-administration was restarted [3]. TSAb-positive Graves' patients with hyperthyroidism were diagnosed on the basis of the history, signs of hyperthyroidism with diffuse goiter, and the laboratory findings, including positive TRAb (TSAb and/or TBII) and elevated fT4 and fT3 with low TSH [1, 2]. The 98 Graves' patients were treated with antithyroid drugs (ATDs). They had been treated with ATD over several years. ATD was discontinued at 6 months after the TSAb-disappearance. After the discontinuation of ATD, the patients had been seen every 1–3 months. When the patients continued to be in euthyroid states and to have negative TSAb and negative TBII for more than 1 year after the ATD-discontinuation, they were considered to be in remission; otherwise, they had recurrence [14]. When they had recurrence, ATD-treatment was restarted. We had followed these 34 TSBAb-positive patients with hypothyroidism and 98 TSAb-positive Graves' patients with hyperthyroidism over 10 years.

### 2.2. Porcine Thyroid Cell Cyclic AMP Production: TSBAb and TSAb

TSBAb and TSAb were measured as before [13, 14]. Cyclic AMP (cAMP) production was determined according to the instruction in commercial assay kit (Yamasa, Chosi, Chiba, Japan). Crude IgG, obtained as PEG (6000) 12.5% precipitated fraction- (final concentration) from 0.2 mL aliquot of test serum, was dissolved in modified Hanks' solution without NaCl. Porcine thyroid cells were incubated with test IgG in 0.25 mL Hanks' solution without NaCl, pH 7.5, containing 1.5% bovine serum albumin, 20 mM Hepes, and 0.5 mM 3-isobutyl-l-methylxanthine. Cyclic AMP production during 5 h incubation at 37°C was measured by radioimmunoassay (RIA), using a commercial kit (Yamasa). To measure TSBAb-activities, crude IgG was incubated with porcine thyroid cells in the presence of 25 *μ*U bTSH (100 mU/L, final concentration), as before [3, 10–13, 15]. Cyclic AMP production during 5 h incubation was measured. TSBAb-activity was expressed as percentage inhibition of bTSH-stimulated cAMP production by test IgG. TSBAb-activity was calculated as follows:TSBAb (%) = [1 − (*c* − *b*)/(*a* − *b*)] × 100 [3, 10–13, 15], where *a*: cAMP generated in the presence of normal IgG and bTSH, *b*: cAMP generated in the presence of normal IgG, and *c*: cAMP generated in the presence of test IgG and bTSH. Test IgG and normal IgG were the 12.5% PEG-precipitated fraction from test serum and normal human serum, respectively. TSBAb, described in this report, corresponds to TSBAb-A in the previous report [13]. TSBAb activities were studied in 95 normal subjects (normal values were less than +40%) [13]. TSBAb activities were more than +40% in all of the TSBAb-positive patients with hypothyroidism. TSAb activity was expressed as percentage cAMP production compared with the mean values for 125 normal subjects (normal values were less than 180%) [1, 2, 14]: TSAb (%) = [*d*/*b*] × 100, where *b*: cAMP generated in the presence of normal IgG, and *d*: cAMP generated in the presence of test IgG.

### 2.3. TSH-Binding Inhibitory Immunoglobulin (TBII)

TBII was measured by radioreceptor assay with a commercial kit (R. S. R. Limited, Cardiff, UK). Assay results were expressed as the percentage inhibition of I^125^-TSH-binding to thyroid plasma membrane as before [1, 2, 5, 14]. Normal values were obtained from 128 normal control subjects and were less than l0% [1, 2, 14].

### 2.4. Statistical Analysis and Others

 All samples were tested in duplicate or triplicate. Statistical analysis was performed using Student's *t*-test or *χ* ^2^-test. *P* values less than 0.05 were considered to be statistically significant. Serum-free T3, -free T4, and TSH were determined by electrochemiluminescence immunoassays (ECLIAs) (Roche Diagnostics, Tokyo, Japan). Normal reference ranges are as follows: fT3 3.5–6.6 nmol/L, fT4 11.6–21.9 pmol/L, and TSH 0.4–4.20 mIU/L. The study plan was reviewed and approved by our institutional review committee. Written informed consent was obtained from the patient prior to publication of this paper.

## 3. Resuls

Serial changes of TSBAb and TSAb over 10 years were studied in 34 TSBAb-positive patients with hypothyroidism and in 98 Graves' patients with hyperthyroidism (Table 1). The 34 TSBAb-positive patients with hypothyroidism (I) were treated with thyroxine (T4) and the 98 TSAb-positive Graves' patients with hyperthyroidism (II) were treated with antithyroid drugs (ATDs). Among the 34 TSBAb-positive patients with hypothyroidism (I), 17 patients (Ia) continued to have persistently positive TSBAb and continued to have hypothyroidism. Half (17) (Ia) of the 34 TSBAb-positive patients continued to have persistently positive TSBAb, continued to have hypothyroidism, and did not recover from hypothyroidism. TSBAb disappeared in 15 (Ib) of the 34 TSBAb-positive patients with hypothyroidism. With the disappearance of TSBAb, recovery from hypothyroidism was seen in 13 (Ib1) (87%) of the 15 patients, in whom TSBAb had disappeared (Ib). Among the 98 TSAb-positive Graves' patients with hyperthyroidism (II), 10 patients (IIa) continued to have persistently positive TSAb and continued to have hyperthyroidism. Ten of the 98 TSAb-positive Graves' patients with hyperthyroidism continued to have persistently positive TSAb. They continued to have hyperthyroidism and did not get remissions of Graves' hyperthyroidism. They continued to take ATD. Complex changes of TSAb were noted in 13 TSAb-positive patients (IIb). One (IIb1) of the 13 patients with complex changes of TSAb got remissions, but the other 12 patients (IIb2) did not. TSAb disappeared in 73 (IIc) (74%) of the 98 TSAb-positive Graves' patients with hyperthyroidism. With the disappearance of TSAb, 60 (IIc1) (82%) of the 73 patients, in whom TSAb had disappeared (IIc), got remissions of Graves' hyperthyroidism. Two TSBAb-positive patients with hypothyroidism developed TSAb-positive Graves' hyperthyroidism (Ic). Two TSAb-positive Graves' patients with hyperthyroidism developed TSBAb-positive hypothyroidism (IId).

Table 2 shows characteristics of the 34 TSBAb-positive patients with hypothyroidism (I) and the 98 TSAb-positive Graves' patients with hyperthyroidism (II). I, Ia, Ib, Ic, II, IIa, IIb, IIc, and IId correspond to those in Table 1. No differences of gender and ages were noted among I, Ia, Ib, Ic, II, IIa, IIb, IIc, and IId. No differences of TSAb-, TSBAb-, and TBII-activities were noted among Ia, Ib, and Ic and among IIa, IIb, IIc, and IId. All of the 34 TSBAb-positive patients with hypothyroidism had strongly positive TSBAb (85–103%, mean ± SD = 92 ± 7%) (Table 2, Ia+Ib+Ic). Some of them had weakly positive TSAb. Their TSAb activity ranged from 92% to 240%. The TSAb activities were 180–240% in 7 (21%) of the 34 TSBAb-positive patients with hypothyroidism and were less than 180% in the other 27 patients (79%). Seven (21%) of the 34 TSBAb-positive patients with hypothyroidism had positive TSAb. TSBAb-positive patients with hypothyroidism had narrow distribution of TSBAb (82–104%, 92 ± 7%) and TSAb (92–240%, 140 ± 9%). All of the 98 Graves' patients with hyperthyroidism had positive TSAb (250–1795%, 775 ± 396%) (Table 2, IIa+IIb+IIc+IId). Some of them had TSBAb. The TSBAb activities were +40–+ 52% in 11 (11%) and were less than +40% in the other 87 patients (89%). Graves' patients with hyperthyroidism had wide distributions of TSAb (250–1795%, 775 ± 396%) and TSBAb (−28–+52%, 10 ± 9%).

### 3.1. 34 TSBAb-Positive Patients with Hypothyroidism (I) (Tables 1 and 2, I)

**Figure 1:**
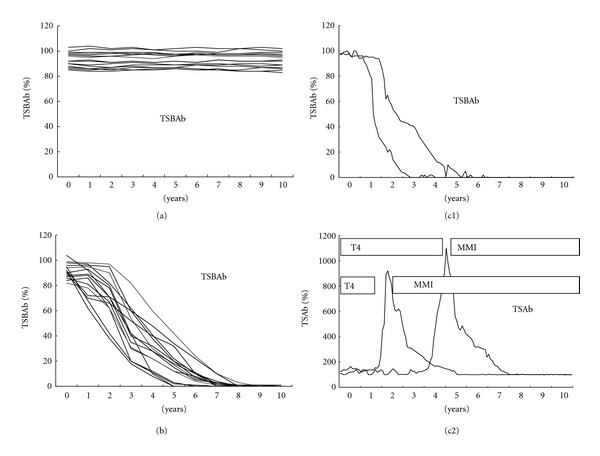
The changes of TSBAb in 34 TSBAb-positive patients with hypothyroidism (Table 1, I). Among the 34 TSBAb-positive patients with hypothyroidism, 17 patients continued to have persistently positive TSBAb and continued to have hypothyroidism (Table 1, Ia) (a). Half of the 34 TSBAb-positive patients continued to have persistently positive TSBAb, continued to have hypothyroidism, and did not recover from hypothyroidism. They continued to take thyroxine (T4). TSBAb disappeared in 15 of the 34 TSBAb-positive patients with hypothyroidism (Table 1, Ib) (b). Recovery from hypothyroidism was noted with the disappearance of TSBAb in 13 (87%) of the 15 patients, in whom TSBAb had disappeared. (c1, c2) show the changes of TSBAb and TSAb, respectively, in the 2 patients with TSBAb-positive hypothyroidism, who developed TSAb-positive Graves' hyperthyroidism (Table 1, Ic). In these 2 patients, TSBAb was dominant initially (c1), and then TSAb became dominant (c2); 2 patients with TSBAb-positive hypothyroidism developed TSAb-positive Graves' hyperthyroidism. Hypothyroidism was treated with thyroxine (T4). Graves' hyperthyroidism was treated with 1-methyl 2-mercapto imidazole (MMI). TSBAb: TSH-stimulation blocking antibody; TSAb: thyroid stimulating antibody.

 All of the 34 TSBAb-positive patients with hypothyroidism had strongly positive TSBAb. Some of them had weakly positive TSAb. TSBAb-positive patients with hypothyroidism had narrow distributions of TSBAb (82–104%, 92 ± 7%) and TSAb (92–240%, 140 ± 9%) (Table 2, Ia+b+c).  Figure 1 shows the changes of TSBAb in the 34 TSBAb-positive patients with hypothyroidism (Table 1, I). Among the 34 TSBAb-positive patients with hypothyroidism (I), 17 (Ia) (Table 1, Ia, Figure 1(a)) continued to have persistently positive TSBAb and continued to have hypothyroidism. Half (17) (Ia) of the 34 TSBAb-positive patients (I) continued to have persistently positive TSBAb, continued to have hypothyroidism, and did not recover from hypothyroidism. They continued to take T4. TSBAb disappeared in 15 (Ib) (Table 1, Ib, Figure 1(b)) of the 34 TSBAb-positive patients (I) with hypothyroidism. With the disappearance of TSBAb, recovery from hypothyroidism was noted in 13 (Ib1) (87%) of the 15 patients, in whom TSBAb had disappeared (Ib).

**Figure 2:**
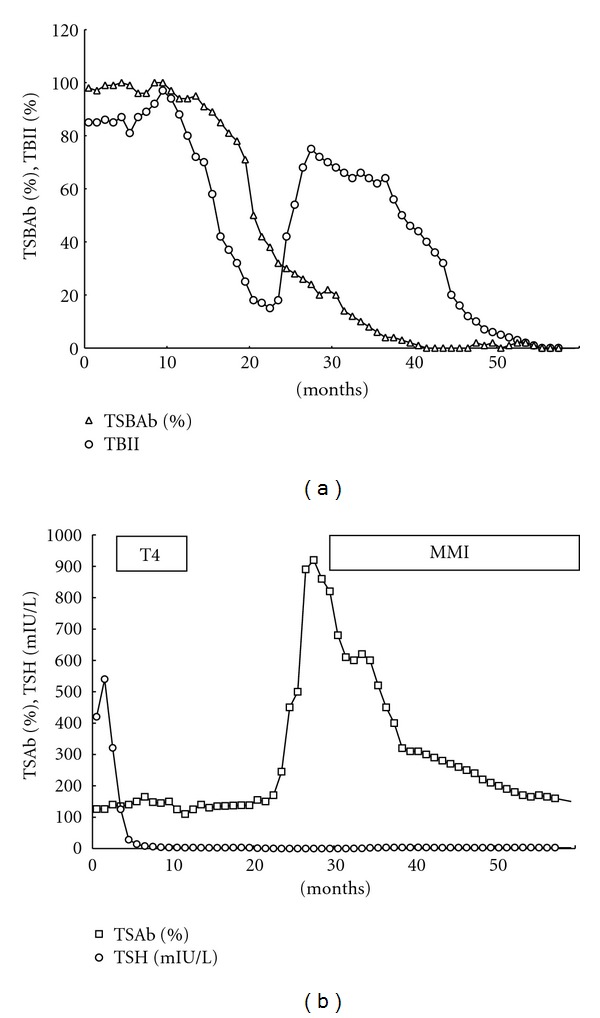
The clinical course of one of the 2 patients, who initially had TSBAb-positive hypothyroidism and then developed TSAb-positive Graves' hyperthyroidism (Table 1, Ic). A 45-year-old woman with TSBAb-positive hypothyroidism developed TSAb-positive Graves' hyperthyroidism. She had TSBAb-positive hypothyroidism ((a), *▵*) with high serum TSH ((b), ∘) and then developed TSAb-positive Graves' hyperthyroidism ((b), **□**) with undetectable serum TSH ((b), ∘). TSBAb was dominant initially ((a), *▵*), and then TSAb became dominant ((b), **□**). TBII (TSH-binding inhibitory immunoglobulin) ((a), ∘) reflects TSBAb- and TSAb-activity. A patient with TSBAb-positive hypothyroidism developed TSAb-positive Graves' hyperthyroidism. She was treated with T4 and then with MMI.

Figures 1(c1) and 1(c2) show the changes of TSBAb and TSAb, respectively, in the 2 patients with TSBAb-positive hypothyroidism, who developed TSAb-positive Graves' hyperthyroidism (Table 1, Ic). In these 2 patients, TSBAb was dominant initially (Figure 1(c1)), and then TSAb became dominant (Figure 1(c2)). These 2 TSBAb-positive patients had hypothyroidism and then developed TSAb-positive Graves' hyperthyroidism. They were treated with T4 and then treated with 1-methyl 2-mercapto imidazole (MMI).  Figure 2 demonstrates the clinical course of one of these 2 patients with TSBAb-positive hypothyroidism, who developed TSAb-positive Graves' hyperthyroidism (Table 1, Ic). A 45-year-old woman with TSBAb-positive hypothyroidism developed TSAb-positive Graves' hyperthyroidism. TSBAb was dominant initially (Figure 2(a)), and then TSAb became dominant (Figure 2(b)). She had TSBAb-positive hypothyroidism with high serum TSH and then developed TSAb-positive Graves' hyperthyroidism with undetectable serum TSH. She was treated with T4 and then treated with MMI. She had a goiter initially and had goitrous autoimmune thyroiditis.

 Among the 34 TSBAb-positive patients with hypothyroidism (Table 1, I), 24 had atrophic autoimmune thyroiditis and 10 had goitrous autoimmune thyroiditis (Table 3(a)). The 34 TSBAb-positive patients with hypothyroidism consisted of 17 patients (a: positive TSBAb persisited), 15 patients (b: TSBAb disappeared), and 2 patients (c: TSBAb → TSAb) (Table 3(a)). All of the 17 (a) patients continued to have positive TSBAb and continued to have hypothyroidism. All of the 17 (a) patients had atrophic autoimmune thyroiditis and none of them had goitrous autoimmune thyroiditis. TSBAb disappeared in the 15 (b) patients: 13 (b1) (87%) of the 15 (b) patients recovered from hypothyroidism and 2 (b2) (13%) of the 15 (b) patients continued to have hypothyroidism. Of the 13 (b1) patients, who recovered from hypothyroidism, 5 had atrophic autoimmune thyroiditis and 8 had goitrous autoimmune thyroiditis. The 2 (b2) patients, who continued to have hypothyroidism, had atrophic autoimmune thyroiditis. Of the 15 (b) patients, in whom TSBAb had disappeared, 7 [5 (b1) + 2 (b2)] had atrophic autoimmune thyroiditis and 8 [8 (b1)] had goitrous autoimmune thyroiditis. Two (c) patients of the 34 TSBAb-positive patients with hypothyroidism developed TSAb-positive Graves' hyperthyroidism had goitrous autoimmune thyroiditis.

Table 3(b) demonstrates recovery from hypothyroidism in the 34 TSBAb-positive patients with hypothyroidism (24 patients with atrophic autoimmune thyroiditis and 10 patients with goitrous autoimmune thyroiditis). Among the 34 TSBAb-positive patients with hypothyroidism, 19 [(17 (a) + 2 (b2)) in Table 3(a)] continued to have hypothyroidism over 10 years and 15 [13 (b1) + 2 (c)] recovered from hypothyroidism (13 (b1) recovered from hypothyroidism and had remissions and 2 (c) recovered from hypothyroidism and developed hyperthyroidism). All of the 19 TSBAb-positive patients with hypothyroidism, who continued to have hypothyroidism [17 (a) + 2 (b2)], had atrophic autoimmune thyroiditis, and none of them had goitrous autoimmune thyroiditis. Fifteen [13 (b1) + 2 (c)] of the 34 TSBAb-positive patients with hypothyroidism recovered from hypothyroidism. Five [5 (b1)] of the 15 patients, who recovered from hypothyroidism, had atrophic autoimmune thyroiditis and the other 10 [8 (b1) + 2 (c)] had goitrous autoimmune thyroiditis. Nineteen (79%) of the 24 TSBAb-positive hypothyroid patients with atrophic autoimmune thyroiditis continued to have hypothyroidism and the other 5 (21%) recovered from hypothyroidism. All (100%) of the 10 TSBAb-positive hypothyroid patients with goitrous autoimmune thyroiditis [8 (b1) + 2 (c)] recovered from hypothyroidism. Significant differences of recovery from hypothyroidism were noted between the patients with goitrous autoimmune thyroiditis and those with atrophic autoimmune thyroiditis (*χ* ^2^ = 17.9, *P*  value < 0.05). All of the 10 TSBAb-positive patients with goitrous autoimmune thyroiditis recovered from hypothyroidism and 19 (79%) of the 24 patients with atrophic autoimmune thyroiditis continued to have hypothyroidism.

### 3.2. 98 TSAb-Positive Graves' Patients with Hyperthyroidism (II) (Tables 1 and 2, II)

**Figure 3:**
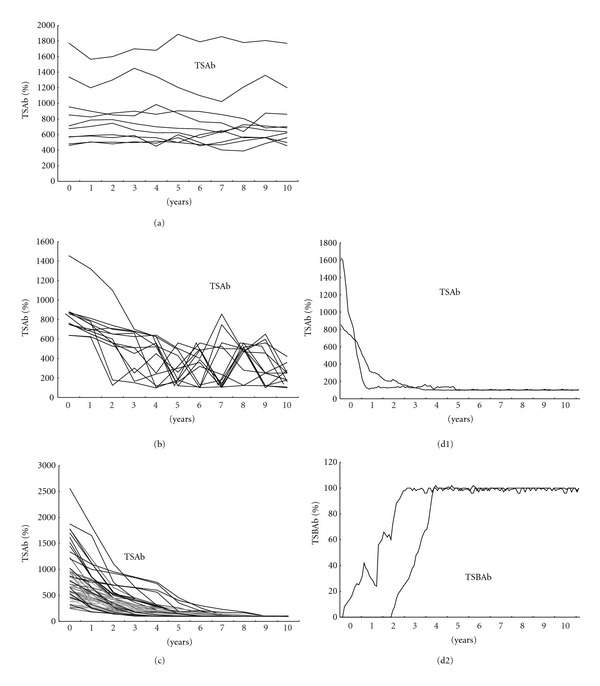
The changes of TSAb in 98 Graves' patients with hyperthyroidism (II) (Table 1, II). Among the 98 Graves' patients with hyperthyroidism, 10 patients continued to have persistently positive TSAb and continued to have hyperthyroidism (Table 1, IIa) (a). Ten of the 98 TSAb-positive Graves' patients with hyperthyroidism continued to have persistently positive TSAb. They continued to have hyperthyroidism and did not get remissions of Graves' hyperthyroidism. They continued to take MMI. Complex changes of TSAb were noted in 13 TSAb-positive patients (Table 1, IIb) (b). One of the 13 patients with complex changes of TSAb got remissions, but the other 12 patients did not get remissions. TSAb disappeared in 73 (74%) of the 98 TSAb-positive Graves' patients with hyperthyroidism (Table 1, IIc) (c). With the disappearance of TSAb, 60 (82%) of the 73 patients, in whom TSAb had disappeared, got remissions of Graves' hyperthyroidism. (d1, d2) show the changes of TSAb and TSBAb, respectively, in the 2 patients with TSAb-positive Graves' hyperthyroidism, who developed TSBAb-positive hypothyroidism (Table 1, IId). In these 2 patients, TSAb was dominant initially (d1), and then TSBAb became dominant (d2). Two patients with TSAb-positive Graves' hyperthyroidism developed TSBAb-positive hypothyroidism. Graves' hyperthyroidism was treated with MMI, and hypothyroidism was treated with T4.

**Figure 4:**
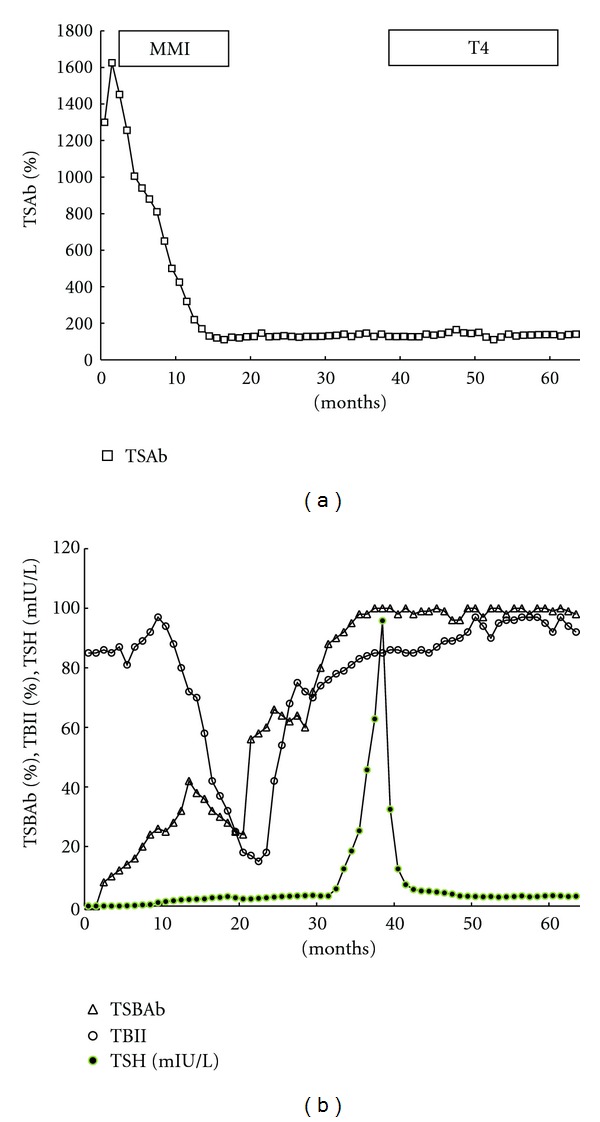
Clinical course of one of the 2 patients, who had TSAb-positive Graves' hyperthyroidism and then developed TSBAb-positive hypothyroidism (Table 1, IId). A 40-year-old woman with TSAb-positive Graves' hyperthyroidism developed TSBAb-positive hypothyroidism. She had TSAb-positive Graves' hyperthyroidism ((a), **□**) with undetectable serum TSH ((b), ●) and then developed TSBAb-positive hypothyroidism ((b), *▵*) with high serum TSH ((b), ●). TSAb was dominant initially ((a), **□**), and then TSBAb became dominant ((b), *▵*). TBII ((b), ∘) reflects TSBAb- and TSAb-activity. A patient with TSAb-positive Graves' hyperthyroidism developed TSBAb-positive hypothyroidism. She was treated with MMI and then with T4.

 All of the 98 Graves' patients with hyperthyroidism had positive TSAb. Some of them had positive TSBAb. Graves' patients with hyperthyroidism had wide distributions of TSAb and TSBAb. Some of the Graves' patients had both positive TSAb and TSBAb.  Figure 3 shows the changes of TSAb in 98 Graves' patients with hyperthyroidism (II) (Table 1, II). Among the 98 Graves' patients with hyperthyroidism, 10 patients continued to have persistently positive TSAb and continued to have hyperthyroidism (IIa) (Figure 3(a)). Ten of the 98 TSAb-positive Graves' patients with hyperthyroidism continued to have positive TSAb and continued to have Graves' hyperthyroidism. They did not get remissions of Graves' hyperthyroidism and continued to take ATD. Complex changes of TSAb were noted in 13 TSAb-positive patients (IIb) (Figure 3(b)). One (IIb1) of the 13 patients with complex changes of TSAb got remissions, but the other 12 patients (IIb2) did not get remissions. TSAb disappeared in 73 (IIc) (74%) of the 98 TSAb-positive Graves' patients with hyperthyroidism (IIc) (Figure 3(c)). With the disappearance of TSAb, 60 (IIc1) (82%) of the 73 patients, in whom TSAb had disappeared (IIc), got remissions of Graves' hyperthyroidism. Figures 3d1 and 3d2 show the changes of TSAb and TSBAb, respectively, in the 2 patients with TSAb-positive Graves' hyperthyroidism, who developed TSBAb-positive hypothyroidism (IId) (Table 1, IId). In these 2 patients, TSAb was dominant initially (Figure 3(d1)), and then TSBAb became dominant (Figure 3(d2)). The 2 patients had TSAb-positive Graves' hyperthyroidism and then developed TSBAb-positive hypothyroidism. They were treated with MMI, and then treated with T4.  Figure 4 demonstrates the clinical course of one of these 2 patients with TSAb-positive Graves' hyperthyroidism, who developed TSBAb-positive hypothyroidism (Table 1, IId). A 40-year-old woman with TSAb-positive Graves' hyperthyroidism developed TSBAb-positive hypothyroidism. TSAb was dominant initially (Figure 4(a)), and then TSBAb became dominant (Figure 4(b)). She had TSAb-positive Graves' hyperthyroidism with undetectable serum TSH and then developed TSBAb-positive hypothyroidism with high TSH. She was treated with MMI and then treated with T4. She had a goiter over 10 years. 

## 4. Discussion

We have reevaluated TSBAb and TSAb in 34 TSBAb-positive patients with hypothyroidism and in 98 TSAb-positive Graves' patients with hyperthyroidism. Half of the 34 TSBAb-positive patients continued to have persistently positive TSBAb, continued to have hypothyroidism and did not recover from hypothyroidism. Ten of the 98 Graves' patients continued to have positive TSAb. They continued to have hyperthyroidism, and did not get remissions of Graves' hyperthyroidism. TSBAb had disappeared in 15 of the 34 TSBAb-positive patients with hypothyroidism. With the disappearance of TSBAb, recovery from hypothyroidism was noted in 13 (87%) of the 15 TSBAb-positive patients. TSAb had disappeared in 73 of the 98 TSAb-positive Graves' patients with hyperthyroidism. With the disappearance of TSAb, 60 (82%) of the 73 TSAb-positive patients got remissions. Two of the 34 TSBAb-positive patients with hypothyroidism developed TSAb-positive Graves' hyperthyroidism. Two of the 98 TSAb-positive Graves' patients with hyperthyroidism developed TSBAb-positive hypothyroidism. TSBAb causes hypothyroidism. TSAb causes Graves' hyperthyroidism. TSBAb and TSAb are TRAb. TSBAb-positive hypothyroidism and TSAb-positive hyperthyroidism may be two aspects of one disease (TRAb disease).

TSBAb blocks TSH-stimulation of the thyroid and causes hypothyroidism. TSAb stimulates the thyroid and causes Graves' hyperthyroidism. Both TSBAb and TSAb block TSH-binding to thyroid cells as TSH receptor antibodies (TRAbs), which have been measured as TSH-binding inhibitory immunoglobulin (TBII) [1–3, 13]. TBII reflects TSBAb- and TSAb-activities. TBII measures the binding of antibody to TSH receptor by competition with radiolabeled TSH and does not distinguish between TSBAb and TSAb. TSBAb is measured as a TSH-stimulation blocking assay and TSAb as a stimulator assay. TSBAb is a blocking antibody [3, 13] and TSAb is a stimulating antibody [1, 2, 13].

TSBAb-activities were expressed as percentage inhibition of TSH-stimulated cAMP production by test IgG [3, 10–13, 15–20]. Two formulas (TSBAb-A and TSBAb-B) have been proposed to calculate TSBAb [3, 10–13]. TSBAb-A was used in the earlier reports [3, 10–13], and TSBAb-B in the later report [13]. TSBAb-A ignores TSAb activity in serum and might give low TSBAb activity. TSBAb-B considers TSAb activity in serum and might give high TSBAb activity. All of the TSBAb-positive patients with hypothyroidism had strongly positive TSBAb-A and TSBAb-B. Both TSBAb-A and TSBAb-B could be used to estimate TSBAb activities [13]. The details were discussed in the previous paper [13]. TSBAb, described in this paper, corresponds to TSBAb-A in the previous paper [13]. TSBAb-A [13] is used as TSBAb in this report.

All of the 34 TSBAb-positive patients with hypothyroidism and all of the 98 TSAb-positive Graves' patients had positive TBII (TRAb). TSBAb and TSAb are TSH-receptor antibodies (TRAbs), which have been measured as TBII. TBII does not distinguish between TSBAb and TSAb. TBII reflects TSBAb- and TSAb-activities [1–3, 13]. All of the 34 TSBAb-positive patients with hypothyroidism had strongly positive TSBAb. Some of them had positive TSAb [13]. All of the 98 Graves' patients had positive TSAb. Some of them had positive TSBAb [13]. TSBAb-positve patients with hypothyroidism had narrow distributions of TSBAb and TSAb, and Graves' patients with hyperthyroidism had wide distributions of TSBAb and TSAb [13]. TSBAb-positive patients with hypothyroidism have strongly positive TSBAb.

TBII reflects TSBAb- and TSAb-activities [1–3, 13]. Some of the TBII-positive patients have hypothyroidism, and the other TBII-positive patients have hyperthyroidism. The former TBII is TSBAb, and the latter TBII is TSAb. The numbers of the former TSBAb-positive patients with hypothyroidism are less than those of the latter TSAb-positive Graves' patients with hyperthyroidism. All of the TSBAb-positive patients with hypothyroidism have high titers of TBII, which is TSBAb [3]. Almost all of the untreated Graves' patients with hyperthyroidism have TBII, which is TSAb [1, 2]. TSBAb- (TRAb-) positive hypothyroidism and TSAb- (TRAb-) positive Graves' hyperthyroidism may be two aspects of one disease (TRAb disease).

Hypothyroidism may result from the production of TSBAb [3]. In 1992, we followed 21 TSBAb-positive patients with hypothyroidism over 10 years and found that with the disappearance of TSBAb, recovery from hypothyroidism was noted in 6 (40%) of the 15 TSBAb-positive patients [3]. Here, we followed 34 TSBAb-positive patients with hypothyroidism over 10 years and found that with the disappearance of TSBAb, recovery from hypothyroidism was noted in 13 (87%) of the 15 patients. The frequency of recovery from hypothyroidism with the disappearance of TSBAb in this paper is much higher than that in the previous one [3]. With the disappearance of TSBAb, recovery from hypothyroidism is observed. The production of TSBAb may subside, producing remissions of hypothyroidism.

It is important to know whether a patient with Graves' disease gets remission or not during ATD treatment. Disappearance of TSAb predicted the remissions of Graves' hyperthyroidism [14]. With the disappearance of TSAb, 36 (82%) of the 44 patients were reported to get remissions in the previous paper [14] and 60 (82%) of the 73 patients are reported to get remissions in this paper. Disappearance of TSAb predicts the remissions of Graves' hyperthyroidism.

Two of the 34 TSBAb-positive patients with hypothyroidism developed TSAb-positive Graves' hyperthyroidism (Ic). Two of the 98 TSAb-positive Graves' patients with hyperthyroidism developed TSBAb-positive hypothyroidism (IId). In the former, TSBAb was dominant initially and then TSAb became dominant. In the latter, TSAb was dominant initially and then TSBAb became dominant. Thyroid function can oscillate between hypothyroidism and hyperthyroidism as TSBAb or TSAb becomes dominant. TSAb and TSBAb can be used to document the functions of TRAb [13]. TBII-positive patients with strongly positive TSBAb have hypothyroidism. TBII-positive patients with positive TSAb have hyperthyroidism. TSBAb-positive patients with hypothyroidism and TSAb-positive Graves' patients with hyperthyroidism may have both TSBAb and TSAb [1, 2, 13, 21–26]. TSBAb-positive patients with hypothyroidism may develop TSAb-positive hyperthyroidism. TSAb-positive Graves' patients with hyperthyroidism may develop TSBAb-positive hypothyroidism. TSBAb and TSAb are TRAb. TSBAb- (TRAb-) positive hypothyroidism and TSAb- (TRAb-) positive hyperthyroidism may be two aspects of one disease (TRAb disease).

In Japan, TRAb has been measured as TBII and TSAb [14]. TSAb is a bioassay, using porcine thyroid cells. We usually measure TSAb, using a commercially available kit [14]. In Japan, TSAb-assay kit is available, but TSBAb-assay kit is not. When a patient has hypothyroidism with elevated TSH and positive TBII, this TBII is thought to be TSBAb. We usually do not measure TSBAb. Practically, when a patient with hypothyroidism has positive TBII, this TBII may be TSBAb. When a patient with hyperthyroidism has positive TBII, this TBII may be TSAb. TSAb and TSBAb can be used to document TRAb-function. TBII, measuring the antibody-binding to the receptor by competition with radio-labeled TSH, does not distinguish between TSAb and TSBAb. A positive TBII result in a patient with hypothyroidism is evidence for the presence of TSBAb. A positive TBII result in a patient with hyperthyroidism is evidence for the presence of TSAb. These bioassays (TSAb and TSBAb) are useful to detect transient neonatal hyperthyroidism and hypothyroidism [10] and are also important to confirm the causes of hyperthyroidism and hypothyroidism [13]. TBII-positive patients may have TSBAb or TSAb. Thyroid function can oscillate between hypothyroidism and hyperthyroidism as TSBAb or TSAb becomes dominant. TSAb and TSBAb can be used to document TRAb-function [13].

There are two forms of autoimmune thyroiditis: atrophic autoimmune thyroiditis and goitrous autoimmune thyroiditis [3]. We followed 34 TSBAb-positive patients with hypothyroidism (24 patients with atrophic autoimmune thyroiditis and 10 with goitrous autoimmune thyroiditis) over 10 years. TSBAb has been found in patients with atrophic autoimmune thyroiditis, and also in patients with goitrous autoimmune thyroiditis [11]. All of the 10 TSBAb-positive patients with goitrous autoimmune thyroiditis recovered from hypothyroidism and 19 (79%) of the 24 with atrophic autoimmune thyroiditis continued to have hypothyroidism. With the disappearance of TSBAb, recovery from hypothyroidism has been seen. TSBAb-positive hypothyroid patients with goitrous autoimmune thyroiditis may recover from hypothyroidism, and those with atrophic autoimmune thyroiditis may continue to have hypothyroidism.

## Figures and Tables

**Table 1:** Changes of TSBAb (TSH-stimulation blocking antibody) and TSAb (thyroid stimulating antibody) over 10 years in 34 TSBAb-positive patients with hypothyroidism and in 98 TSAb-positive Graves' patients with hyperthyroidism.

	(I) 34 TSBAb-positive patients with hypothyroidism		34
Ia: Positive TSBAb persisted	Continued to have hypothyroidism	17	17
Ib: TSBAb disappeared	Ib1: Recovered from hypothyroidism	13	15
	Ib2: Continued to have hypothyroidism	2	
Ic: TSBAb → TSAb	TSBAb-positive hypo → Graves' hyper	2	2

	(II) 98 TSAb-positive Graves' patients with hyperthyroidism		98

IIa: Positive TSAb persisted	Continued to have Graves' hyperthyroidism	10	10
IIb: Complex changes of TSAb	IIb1: Remission	1	13
	IIb2: Recurrence	12	
IIc: TSAb disappeared	IIc1: Remission	60	73
	IIc2: Recurrence	13	
IId: TSAb → TSBAb	Graves' hyper → TSBAb-positive hypo	2	2

Numbers of the patients are shown.

Serial changes of TSBAb and TSAb over 10 years were studied in 34 TSBAb-positive patients with hypothyroidism (I) and in 98 TSAb-positive Graves' patients with hyperthyroidism (II). The 34 TSBAb-positive patients with hypothyroidism were treated with thyroxine (T4) and the 98 TSAb-positive Graves' patients with hyperthyroidism were treated with antithyroid drugs (ATDs). Half (17) (Ia) of the 34 TSBAb-positive patients with hypothyroidism (I) continued to have positive TSBAb and continued to have hypothyroidism. Ten (IIa) of the 98 TSAb-positive Graves' patients with hyperthyroidism (II) continued to have positive TSAb and continued to have Graves' hyperthyroidism. With the disappearance of TSBAb, recovery from hypothyroidism was noted in 13 (Ib1) (87%) of the 15 patients, in whom TSBAb had disappeared (Ib). With the disappearance of TSAb, remissions of Graves' hyperthyroidism were noted in 60 (IIc1) (82%) of the 73, in whom TSAb had disappeared (IIc). Two of the 34 TSBAb-positive patients with hypothyroidism developed TSAb-positive Graves' hyperthyroidism (Ic), and two of the 98 TSAb-positive Graves' patients with hyperthyroidism developed TSBAb-positive hypothyroidism (IId).

**Table 2:** Characteristics of the 34 TSBAb-positive patients with hypothyroidism and the 98 TSAb-positive Graves' patients with hyperthyroidism.

	Number of patients	Gender	Age (years)	Before treatment		
		Men/Women		TSBAb (%)	TSAb (%)	TBII (%)
	(I) 34 TSBAb-positive patients with hypothyroidism					

Ia	17	5/12	42 ± 17	**94 ± 6**	146 ± 10	**95 ± 5**
Ib	15	4/11	45 ± 16	**90 ± 9**	136 ± 8	**92 ± 7**
Ic	2	1/1	38, 45	**98, 97**	100, 98	**96, 95**
Ia+Ib+Ic	34	10/24	43 ± 18	**92 ± 7**	140 ± 9	**94 ± 7**

	(II) 98 TSAb-positive Graves' patients with hyperthyroidism					
IIa	10	3/7	40 ± 16	9 ± 8	**839 ± 421**	**76 ± 15**
IIb	13	3/10	42 ± 17	10 ± 11	**846 ± 195**	**68 ± 16**
IIc	73	18/55	44 ± 16	10 ± 10	**746 ± 390**	**56 ± 18**
IId	2	0/2	40, 48	2, 5	**1625, 852**	**76, 58**
IIa+IIb+IIc+IId	98	24/74	43 ± 17	10 ± 9	**775 ± 396**	**57 ± 17**

Values are means ± SD. I, Ia, Ib, Ic, II, IIa, IIb, IIc, and IId correspond to those in Table 1. No differences of gender and ages were noted among I, Ia, Ib, Ic, II, IIa, IIb, IIc, and IId. No differences of TSAb-, TSBAb-, and TBII-activities were noted among Ia, Ib, and Ic and among IIa, IIb, IIc, and IId.

All of the 34 TSBAb-positive patients with hypothyroidism had strongly positive TSBAb (85–103%, mean ± SD = 92 ± 7%) (Ia+Ib+Ic). Some of them had weakly positive TSAb. Their TSAb activity ranged from 92% to 240%. The TSAb activities were 180–240% in 7 (21%) of the 34 TSBAb-positive patients with hypothyroidism and were less than 180% in the other 27 patients (79%). Seven (21%) of the 34 TSBAb-positive patients with hypothyroidism had positive TSAb. TSBAb-positive patients with hypothyroidism had narrow distribution of TSBAb (82–104%, 92 ± 7%) and TSAb (92–240%, 140 ± 9%). All of the 98 Graves' patients with hyperthyroidism had positive TSAb (250–1795%, 775 ± 396%) (IIa+IIb+IIc+IId). Some of them had TSBAb. The TSBAb activities were +40–+52% in 11 (11%) and were less than +40% in the other 87 patients (89%). Graves' patients with hyperthyroidism had wide distributions of TSAb (250–1795%, 775 ± 396%) and TSBAb (−28–+52%, 10 ± 9%).

**Table:** (a) Atrophic autoimmune thyroiditis (atrophic) or goitrous autoimmune thyroiditis (goitrous) in the 34 TSBAb-positive patients with hypothyroidism

		34 TSBAb-positive patients with hypothyroidism (I in Table 1)^†^		34	
				Atrophic (24)	Goitrous (10)
a: Positive TSBAb persisted (Ia)			17	17	0
b: TSBAb disappeared (Ib)	15	b1: recovered (Ib1)	13	5	8
		b2: hypothyroid (Ib2)	2	2	0
c: TSBAb → TSAb (Ic)			2	0	2

Numbers of the patients are shown. (I in Table 1)^†^correspond to those in Table 1

Among the 34 TSBAb-positive patients with hypothyroidism (Table 1, I), 24 had atrophic autoimmune thyroiditis and 10 had goitrous autoimmune thyroiditis. The 34 TSBAb-positive patients with hypothyroidism consisted of 17 patients (a: positive TSBAb persisited), 15 patients (b: TSBAb disappeared), and 2 patients (c: TSBAb → TSAb). All of the 17 (a) patients continued to have positive TSBAb and continued to have hypothyroidism. All of the 17 (a) patients had atrophic autoimmune thyroiditis and none of them had goitrous autoimmune thyroiditis. TSBAb disappeared in the 15 (b) patients: 13 (b1) (87%) of the 15 (b) patients recovered from hypothyroidism and 2 (b2) (13%) of the 15 (b) patients continued to have hypothyroidism. Of the 13 (b1) patients, who recovered from hypothyroidism, 5 had atrophic autoimmune thyroiditis and 8 had goitrous autoimmune thyroiditis. The 2 (b2) patients, who continued to have hypothyroidism, had atrophic autoimmune thyroiditis. Of the 15 (b) patients, in whom TSBAb had disappeared, 7 [5 (b1) + 2 (b2)] had atrophic autoimmune thyroiditis and 8 [8 (b1)] had goitrous autoimmune thyroiditis. Two (c) patients of the 34 TSBAb-positive patients with hypothyroidism who developed TSAb-positive Graves' hyperthyroidism who had goitrous autoimmune thyroiditis.

**Table:** (b) Recovery from hypothyroidism in the patients with atrophic autoimmune thyroiditis (atrophic) and in those with goitrous autoimmune thyroiditis (goitrous)

	Atrophic (24)	Goitrous (10)		
Continued to have hypothyroidism	19 (79%) [17 (a) + 2 (b2)]*	0 (0%)	19	
Recovered from hypothyroidism	5 (21%) [5 (b1)]*	10 (100%) [8 (b1) + 2 (c)]*	15	*χ* ^2^ = 17.9 *P* value < 0.05

	24 (100%)	10 (100%)	34	

Numbers (%) of the patients are shown. [ ]*corresponds to Table 3(a).

Among the 34 TSBAb-positive patients with hypothyroidism (Table 1, I), 24 had atrophic autoimmune thyroiditis and 10 had goitrous autoimmune thyroiditis. Among the 34 TSBAb-positive patients with hypothyroidism, 19 [(17 (a) + 2 (b2)] (Table 3(a)) continued to have hypothyroidism over 10 years and 15 [13 (b1) + 2 (c)] recovered from hypothyroidism [13 (b1) recovered from hypothyroidism and had remissions and 2 (c) recovered from hypothyroidism and developed hyperthyroidism]. All of the 19 TSBAb-positive patients with hypothyroidism, who continued to have hypothyroidism [17 (a) + 2 (b2)], had atrophic autoimmune thyroiditis, and none of them had goitrous autoimmune thyroiditis. Fifteen [13 (b1) + 2 (c)] of the 34 TSBAb-positive patients with hypothyroidism recovered from hypothyroidism. Five [5 (b1)] of the 15 patients, who recovered from hypothyroidism, had atrophic autoimmune thyroiditis and the other 10 [8 (b1) + 2 (c)] had goitrous autoimmune thyroiditis. Nineteen (79%) of the 24 TSBAb-positive hypothyroid patients with atrophic autoimmune thyroiditis continued to have hypothyroidism and the other 5 (21%) of them recovered from hypothyroidism. All of the 10 TSBAb-positive hypothyroid patients with goitrous autoimmune thyroiditis [8 (b1) + 2 (c)] recovered from hypothyroidism. Significant differences of recovery from hypothyroidism were noted between the patients with goitrous autoimmune thyroiditis and those with atrophic autoimmune thyroiditis (*χ* ^2^  = 17.9, *P*  value < 0.05). All (100%) of the 10 TSBAb-positive patients with goitrous autoimmune thyroiditis recovered from hypothyroidism and 19 (79%) of the 24 patients with atrophic autoimmune thyroiditis continued to have hypothyroidism.
